# 

**Published:** 1890-02-08

**Authors:** 


### NEW OFFICES IN THE STRAND.

The Hospital *is now published at* **140, Strand, W.C.** *It has been found desirable to establish* The Hospital *in this central and convenient locality, owing to the rapid extension of the business of this paper of late.*

### NOTICE TO CORRESPONDENTS.

All MS., Letters, Books for Review, and other matters intended for the Editor should be addressed The Editor, The Lodge, Porchester Square, London, W.

**Notice to Advertisers and Subscribers.**

All Advertisements, Orders for Copies of The Hospital, and Business Communications should be addressed to The Publisher, *not to the Editor*, The Hospital, 140, Strand, W.C.

**Bound Volumes of "The Hospital."**

Vol. VI., containing full page portraits of T.R.H. the Prince and Princess of Wales, and Miss Florence Nightingale, is now ready, 4s. post free. Cases for binding may be had of the Publisher, at 1s. 6d. each.

**To Residents, Secretaries, and Matrons.**

The Editor will be glad to have the Regulations and each Report as issued by Asylums, Hospitals, Convalescent and Nursing Institutions, as well as those of other Charities, and an early copy in duplicate of all Appeals and Festival Notices, etc., addressed to *The Editor, The Lodge, Porchester Square, London, W.* Any superintendent, secretary, matron, or other chief official who regularly sends such information may be registered, on application to the Publisher, to receive free of cost a cover for binding The Hospital in half-yearly volumes.

### Scraps and Gleanings.

Pneumonia is very prevalent in New York.

New York is to have a new Hebrew Hospital.

Influenza is still raging in some country districts.

Cholera is said to be approaching through Mesopotamia.

Influenza prevails, in an epidemic form, at Buenos Ayres.

The lectures at the Royal College of Surgeons commence on February 10th.

The death is announced of Mr. B. V. Sortain, student at St. George's Hospital.

There is a new hospital in Baltimore, the Garrett Free Hospital for Children.

The death is announced of Dr. F. W. G. Morgan, house surgeon to Bradford Infirmary.

Nottingham Borough Lunatic Asylum has had a new wing added, capable of holding 280 patients.

A Ladies' Medical Society has been formed in connection with the Woman's Medical College of New York.

The Lord Provost moved the adoption of the annual report of Glasgow Royal Infirmary on January 27th.

Birmingham Children's Hospital does good work, but ever since 1877 the subscription list has been decreasing.

Manchester proposes to have a large hospital for imbecile and epileptic cases. The cost will probably be £70,000.

The last returns of the Metropolitan Asylums Board show that fever and febrile diseases are steadily decreasing in London.

It is stated that Mr. John Horniman, of Croydon, has given £25,000 to wards establishing a seaside convalescent home for children.

Dr. Ogilvy, B.A., M.B., B.C.H., B.A.O., &c., Trinity College, Dublin, has been appointed house surgeon to the Bristol Eye Hospital.

In spite of the muzzling order there were 13 cases of rabies last quarter, as against three in the corresponding quarter of 1888.

Surgeon-General G. M. Slaughter has been appointed Principal Medical Officer on the Staff of the Southern District at Portsmouth.

St. Thomas's Hospital has received presents of game from the Queen, the Prince of Wales, and from Colonel Charles Seely, of the Isle of Wight.

Brighton Throat and Ear Hospital has successfully reached the age of 12 years; 1,147 patients were treated during the last twelve months.

The Duchess of Fife has consented to open a bazaar to be held in the grounds of University College, in June next, on behalf of the funds of University Hospital.

Last year 61,027 deaths took place in England and Wales from the principal zymotic diseases—diarrhœa, measles, whooping-cough, fever, diphtheria, small-pox.

An excellent bazaar has been held at Beverley Cottage Hospital. The matron, Miss Kirwood, has also entertained a large company at tea; her energy is commendable.

The East London Nursing Society has received £50 from S. S. F., and £54 from D. C.; Mrs. Frederick Beer has also promised £36 annually for the district nurse of Wapping.

The Duchess of Albany has accepted the presidency of the Hammersmith Centre of the St. John's Ambulance Association, and the Committee are exceedingly honoured and gratified.

The students of Basle have been protesting against the medical lectures being opened to women. Their objection appeared to be principally that a large proportion of the ladies were foreigners.

At a recent meeting of the principals and governors of the Sunderland Orphan Asylum, a vote of thanks was accorded to Mrs. Laing for collecting £12,000 to pay off the debt on the institution.

It appears by the last official sanitary report of Burmah that the Burmese are very reluctant to avail themselves of European medical remedies offered by the Government dispensaries and hospitals. They rarely enter a hospital.

The first festival dinner in aid of the East End Mothers' Home was held on January 29th, at the Hôtel Métropole, the Earl of Aberdeen presiding. The lady superintendent, Mrs. Ashton Warner, announced a list of donations and subscriptions amounting to £700.

H.E. Lady Zetland and the Countess Grosvenor visited the Adelaide Hospital on the 24th ult., and inspected the Nurses' Home in connection with it. Lady Zetland expressed great pleasure at the admirable arrangements and home-like appearance of this valuable institution.

At the twenty-second anniversary banquet of the French Hospital and Dispensary in Leicester Square, to be held on the 15th inst., in the Hotel Metropole, M. Waddington, the French Ambassador, will preside, supported by Sir Henry Isaacs (the Lord Mayor) and the sheriffs of London.

At a meeting of the Hampshire County Council a report was submitted which showed that influenza had been seriously felt at the County Asylum. There had been 192 cases; 65 male, 75 female patients, and 54 employés; and, unfortunately, one nurse and a patient had died therefrom.

The Registrar-General points out that there are now something like 600,000 people alive in England and Wales whose deaths would have been registered if the rate of mortality had continued as it was between 1871 and 1881. This is nearly equal to the whole white population of New Zealand.

A supporter of the Mission to Deep Sea Fishermen has kindly offered a contribution of £1,000, on condition that a similar sum is raised from the public in order to enable the council to wipe off existing liabilities, and to ensure the despatch of the fully-equipped new mission and hospital ship *Albert*.

Dr. King, an American lady, occupies the position of Physician-in-Ordinary to Count Li, one of the most distinguished statesmen in China. She also possesses a valuable practice in Shanghai, where some of her surgical operations have excited the admiration of her medical brethren. It appears that there is a wide field of employment open to women doctors in the Chinese Empire.

